# Identification and characterization of a *Streptococcus equi* ssp*. zooepidemicus* immunogenic GroEL protein involved in biofilm formation

**DOI:** 10.1186/s13567-016-0334-0

**Published:** 2016-04-18

**Authors:** Li Yi, Yang Wang, Zhe Ma, Hui-Xing Lin, Bin Xu, Daniel Grenier, Hong-Jie Fan, Cheng-Ping Lu

**Affiliations:** Key Lab of Animal Bacteriology, Ministry of Agriculture, Nanjing Agricultural University, Nanjing, China; College of Life Science, Luoyang Normal University, Luoyang, China; College of Animal Science and Technology, Henan University of Science and Technology, Luoyang, China; Groupe de Recherche En Écologie Buccale (GREB), Faculté de Médecine Dentaire, Université Laval, Québec City, QC Canada; Jiangsu Co-innovation Center for the Prevention and Control of Important Animal Infectious Diseases and Zoonoses, Yangzhou, China

## Abstract

*Streptococcus equi* ssp*. zooepidemicus* (*S. equi* spp. *zooepidemicus*) is an opportunistic pathogen that causes major economic losses in the swine industry in China and is also a threat for human health. Biofilm formation by this bacterium has been previously reported. In this study, we used an immunoproteomic approach to search for immunogenic proteins expressed by biofilm-grown *S. equi* spp. *zooepidemicus*. Seventeen immunoreactive proteins were found, of which nine common immunoreactive proteins were identified in planktonic and biofilm-grown bacteria. The immunogenicity and protective efficacy of the *S. equi* spp. *zooepidemicus* immunoreactive GroEL chaperone protein was further investigated in mice. The protein was expressed in vivo and elicited high antibody titers following *S. equi* spp. *zooepidemicus* infections of mice. An animal challenge experiment with *S. equi* spp. *zooepidemicus* showed that 75% of mice immunized with the GroEL protein were protected. Using in vitro biofilm inhibition assays, evidence was obtained that the chaperonin GroEL may represent a promising target for the prevention and treatment of persistent *S. equi* spp. *zooepidemicus* biofilm infections. In summary, our results suggest that the recombinant GroEL protein, which is involved in biofilm formation, may efficiently stimulate an immune response, which protects against *S. equi* spp. *zooepidemicus* infections. It may therefore be a candidate of interest to be included in vaccines against *S. equi* spp. *zooepidemicus* infections.

## Introduction

*Streptococcus equi* ssp*. zooepidemicus* (*S. equi* spp. *zooepidemicus*) is an opportunistic pathogen that causes important economic losses in the swine industry in China and is also a threat for human health [1]. It is indeed a major problem in China and the development of an effective vaccine is vital to circumvent the significant economic impact to the industry. Previous studies have investigated different immunogenic proteins of planktonically grown *S. equi* spp. *zooepidemicus*, including secreted and surface-associated proteins [2–4]. Mao et al. [3, 4] used the immunoproteomic technology to study the convalescent serum of mini-pigs and identified 12 membrane-associated proteins, 13 cell wall-associated proteins, and seven extracellular proteins in *S. equi* spp. *zooepidemicus*. Based on enzymatic shaving and Western blot analyses, Wei et al. [2] described five novel membrane-associated vaccine candidate proteins.

We recently reported that *S. equi* spp. *zooepidemicus* can form biofilms [5]. Given that bacteria within biofilms have an increased resistance to the host defence system and antibiotics, infections caused by biofilm-producing bacteria are frequently chronic, recurrent, and resistant to antibacterial chemotherapy [6]. Immunoprophylaxis and immunotherapy targeting biofilm-related proteins are promising new approaches for the prevention and treatment of biofilm-associated infections [7]. The immunoproteomic technology has been successfully used to identify relevant bacterial antigens for the development of new vaccines [8, 9]. This technique might also be useful to increase our understanding of the molecular mechanisms that control biofilm formation by *S. equi* spp. *zooepidemicus*.

In the present study, immunogenic proteins expressed by biofilm-grown *S. equi* spp. *zooepidemicus* were identified using an immunoproteomic approach. The chaperonin GroEL was found to be an immunogenic protein in both biofilm- and planktonic-grown *S. equi* spp. *zooepidemicus*. The immunogenicity of the recombinant GroEL protein, the protection rate of GroEL-immunized mice, and the capacity of anti-GroEL antibodies to inhibit biofilm production by *S. equi* spp. *zooepidemicus* in vitro were also investigated.

## Materials and methods

### Ethical statements

All the animal experiments were performed with the approval of the Laboratory Animal Monitoring Committee of Jiangsu Province (SYXK (SU) 2011-0036). All efforts were made to minimize animal suffering and to reduce the number of animals used.

### Bacteria and culture conditions

*Streptococcus equi* ssp. *zooepidemicus* strain ATCC 35246 was initially isolated from a diseased pig in Sichuan Province, China, in 1976. The strain, which was purchased from the American Type Culture Collection (ATCC), was cultured in Todd-Hewitt broth (THB, Oxoid) and on THB agar plates. Biofilms of *S. equi* spp. *zooepidemicus* were prepared by growing bacteria in THB medium supplemented with 1% human fibrinogen (Sigma) in polystyrene Petri dishes at 37 °C for 24 h, as described previously [5]. Quantification of biofilm production was carried out by a microtiter plate assay using crystal violet staining. *S. equi* spp. *zooepidemicus* planktonic cells grown at 37 °C for 24 h in Erlenmeyer flasks containing the above culture medium were used as the control.

### Whole cell protein sample extraction

Bacterial proteins were prepared as described previously [5, 10]. Briefly, following biofilm formation, the medium containing free-floating bacteria was poured off, and the wells were washed three times with sterile PBS to remove loosely attached bacteria. The biofilms were detached by scraping, were suspended in 50 mM Tris-HC1 (pH 7.5), and were sonicated for 5 min. The sonicated biofilms were pelleted by centrifugation at 8000 × *g* for 5 min at 4 °C. The pellets were washed twice in 50 mM Tris-HC1 (pH 7.5) and were then resuspended in buffer (50 mM Tris–HCl, pH 7.5, 3 mM MgCl_2_, 50% sucrose) containing 1000 U/mL of mutanolysin (Sigma) and were incubated for 90 min at 37 °C. Spheroplasts were collected and were resuspended by sonication on ice (100 W, 90 cycles, 5 s on and 10 s off) in sonication buffer (7 M urea, 2 M thiourea, 4% 3-[(3-cholamidopropyl) dimethylammonio]-1- propanesulfonate (CHAPS), and 65 mM dithiothreitol (DTT,GE Healthcare). Spheroplasts were then incubated at 25 °C for 30 min, following which cell debris and unbroken cells were removed by centrifugation at 10 000 × *g* for 30 min at 25 °C. The supernatants were mixed with 10% Trichloroacetic acid (TCA) and were incubated in ice water for 30 min. The precipitated proteins were pelleted by centrifugation at 10 000 × *g* for 10 min at 4 °C and were washed twice with pre-chilled acetone. The final pellet was air-dried, dissolved in sample preparation solution (7 M urea, 2 M thiourea, 4% CHAPS, 65 mM DTT), incubated for 30 min at 25 °C (vortexed every 10 min), and centrifuged at 10 000 × *g* for 20 min at 25 °C. Prior to rehydration, the supernatants were treated with 2-D Clean-up kits (GE Healthcare) to remove contaminants that may interfere with the electrophoresis. The protein content was determined using PlusOne 2-D Quant kits (GE Healthcare).

### 2-D gel electrophoresis

The 2-DE (two-dimensional gel electrophoresis) was performed using the immobiline/polyacrylamide system. The isoelectric focusing (IEF) was performed using IPG DryStrips (13 cm; IPGphor; GE Healthcare). Protein samples (200 μg) were applied to the IPG strips using the in-gel sample rehydration technique according to the manufacturer’s instructions. The IEF was performed in a protein IEF cell (GE Healthcare) using a stepwise voltage gradient to 80 kVh. The strips were equilibrated for 2 × 15 min in equilibration buffer (6 M urea, 2% SDS, 30% glycerol, 50 mM Tris–HCl, pH 8.8) supplemented with 1% DTT and 4% iodoacetamide prior to running the second dimension. The SDS-PAGE was carried out vertically on 12.5% polyacrylamide gels using an Ettan DALT II system (GE Healthcare). Resolved proteins were routinely stained with Coomassie Brilliant Blue G-250 for protein identification purposes. All experiments were performed in triplicate. The reproducibility of the 2-DE was verified by running the same samples at least three times on separate gels. Three replicate gels from three independent experiments were run for each growth condition. The gels were compared using Image Master Platinum 5.0 software (GE Healthcare).

### Preparation of convalescent sera

The pathogen-free mini-pigs used in this study had no history of *S. equi* spp. *zooepidemicus* infection and were found to be negative for antibodies against *S. equi* spp. *zooepidemicus* ATCC 35246 whole cells, as determined by an enzyme-linked immunosorbent assay (ELISA). Swine convalescent sera were obtained from the pigs artificially infected with live *S. equi* spp. *zooepidemicus* ATCC 35246. Pre-infection sera were used as a negative control. The protocol of Zhang and Lu [11] was used for the immunization procedure and immunogen preparation. Pigs were intramuscularly injected twice at 3-week interval with 1.0 × 10^9^ CFU (Colony-Forming Units) of *S. equi* spp. *zooepidemicus*. Swine sera were collected 7 days after the booster injection, and serum IgG antibody titers were determined using a whole cell ELISA [3]. Wells of the microplate were coated with formaldehyde-inactive *S. equi* spp. *zooepidemicus* (1.0 × 10^6^ cells), and blocked with 5% skim milk. Two-fold serial dilutions (from 1:500 to 1:16 000) of the sera were added and the plate was incubated for 2 h. Following incubation in the presence of horseradish peroxidase (HRP)-conjugated goat anti-mouse IgG as the secondary antibody, the color was developed by adding 3,3′,5,5′-tetramethylbenzidine (TMB) substrate (Beyotime Institute, China). Color development was recorded by reading the absorbance of 450 nm using a microtiter plate reader (Bio-Rad, USA). Sera with high titers were selected for subsequent experiments.

### Western blotting

Protein samples from each SDS-PAGE gel were transferred onto polyvinylidene difluoride (PVDF) membranes (GE Healthcare) using a semi-dry blotting apparatus (TE77, GE Healthcare) for 2 h at 0.65 mA/cm^2^. After the transfer, membranes were blocked by incubation for 2 h in 100 mM Tris–HCl, 150 mM NaCl, and 0.05% Tween-20 (TBST) containing 5% skim milk. The blocked membranes were incubated with sera from convalescent mini-pigs (1:1000 dilution) for 2 h at room temperature with gentle agitation. They were then washed three times with TBST (10 min per wash) and were incubated (1 h with gentle agitation) with horseradish peroxidase-labeled Staphylococcal protein A (Boster, China) at a dilution of 1:5000 in blocking solution. The membranes were washed as before, and were incubated with 3,3′-diaminobenzidine (Tiangen, China) until the optimum color was obtained. This analysis was repeated three times for each sample.

### Mass spectrometric analysis of protein spots and database searches

The Coomassie Brilliant Blue-stained spots corresponding to the immunoreactive proteins were excised from the 2-D gels and were sent to Shanghai Applied Protein Technology Co. Ltd for tryptic in-gel digestion, Matrix-Assisted Laser Desorption/Ionization Time of Flight Mass Spectrometry (MALDI-TOF–MS), and MALDI-TOF/TOF–MS. The MALDI-TOF–MS and MALDI-TOF/TOF–MS acquisition data were used in a combined search against the NCBInr protein database using MASCOT (Matrix Science), with parameter sets for trypsin digestion, one max missed cleavage, variable modification of oxidation (M), and a peptide mass tolerance for monoisotopic data of 100 ppm. The MASCOT server was originally used to search the NCBInr for peptide mass fingerprinting (PMF). The criteria used to accept protein identifications were based on PMF data, including the extent of sequence coverage, the number of peptides matched, and the score of probability. Protein identification was assigned when the following criteria were met: at least four matching peptides and sequence coverage greater than 15%.

### Confirmation of the immunogenicity of selected proteins

The GroEL chaperone protein was selected for molecular cloning and its immunogenicity was confirmed. The primer pairs of GroEL-S (5′-CGCGAATTCGATATTTTGGCGGATACCGT-3′) and GroEL-A (5′-CC CTCGAGAGCAGGCTCTGGCTTAGTGG-3′) were designed to express the recombinant protein based on the gi number (Table 1). More specifically, *GroEL* PCR product was cloned into the pET28a expression vector as described in a previous study [12]. The resulting plasmid was used to transform *Escherichia coli* DH5α. (TaKaRa, China). Expression of the recombinant protein was induced in *E. coli* BL21(DE3) (TaKaRa, China) at 37 °C for 4 h with 0.1 mM IPTG (Isopropyl β-d-Thiogalactoside). The protein was purified by HisTrap FF column (GE Healthcare, USA) and purity was analyzed by SDS-PAGE [13]. The immunogenicity of the GroEL protein was confirmed by Western blot analysis using pig convalescent sera directed against the *S. equi* spp. *zooepidemicus* ATCC 35246 strain.

**Table 1:** Immunoproteins identified by MALDI-TOF/TOF MS

Spot no.	Protein identified ^a^	BLASTX similarity matched protein/species/identity score	Theoretical MW*/*pI ^b^	Experimental MW*/*pI	MASCOT score ^c^	No. of peptides matched ^d^	Coverage (%)^e^
BF1	gi|338846659	DNA polymerase III delta subunit	39 994/5.80	22 000/5.70	252	19	55
BF2	gi|195977415	elongation factor G	76 540/4.83	76 000/4.80	232	20	40
BF3	gi|195978009	pyruvate kinase	54 638/5.09	55 000/5.10	374	36	62
BF4	gi|338847723	Transketolase	71 341/5.07	71 000/5.09	272	11	21
BF5	gi|338846410	chaperone protein DnaK (heat shock protein 70)	65 045/4.64	60 000/4.60	269	22	36
BF6	gi|225867742	60 kDa chaperonin GroEL	56 876/4.70	56 000/4.60	575	34	60
BF7	gi|195978262	30S ribosomal protein S1	43 801/4.93	43 000/5.00	172	16	46
BF8	gi|338848099	inosine-5′-monophosphate dehydrogenase	53 007/5.48	53 000/5.60	257	29	63
BF9	gi|338846419	3-oxoacyl-(acyl-carrier-protein) synthase II	43 738/5.44	40 000/5.40	164	17	52
BF10	gi|338847987	adenylosuccinate synthetase	47 501/5.47	47 000/5.60	155	18	49
BF11	gi|225869251	phosphoglycerate kinase	42 184/4.96	42 000/4.90	120	14	38
BF12	gi|225867788	glucose-6-phosphate isomerase	49 490/4.88	42 000/4.80	207	23	48
BF13	gi|225869252	glyceraldehyde-3-phosphate dehydrogenase	37 025/5.57	40 000/5.00	243	24	58
BF14	gi|338846346	pyridine nucleotide-disulfide oxidoreductase	47 715/5.30	47 000/5.30	191	21	56
BF15	gi|338847824	oligopeptide ABC transporter periplasmic oligopeptide-binding protein OppA	72 742/5.56	72 000/5.60	244	25	49
BF16	gi|338847405	Elongation factor-Tu	44 545/4.89	21 000/5.30	109	9	22
BF17	gi|225867729	elongation factor Ts	37 263/4.86	30 000/4.40	193	20	59

^a^gi number in NCBI.

^b^Theoretical MW and pI was calculated using compute pI/MW [38].

^c^MASCOT score obtained for the peptide mass fingerprint (PMF). The significance threshold was 70.

^d^Number of peptides that match the predicted protein sequence.

^e^Percentage of predicted protein sequence covered by matched peptides.

### Mouse vaccination and determination of protection efficacy

Thirty-two ICR (Institute of Cancer Research) mice (4-week-old females) were randomly assigned to four groups of eight mice each. The mice in Group 1 were intramuscularly injected with 0.2 mL of recombinant GroEL protein (rGroEL; 100 µg/mL) mixed with an equivalent volume of MONTANIDE ISA 206 VG (SEPPIC, France). The mice in Group 2 were intramuscularly injected with 0.2 mL of formaldehyde-inactivated *S. equi* spp. *zooepidemicus* ATCC35246 vaccine (1 × 10^9^ cfu/mL) combined with MONTANIDE ISA 206 VG and served as positive controls. The second booster from groups 1 and 2 were intramuscularly injected with the recombinant GroEL protein and whole-cell vaccine at day 14, respectively. The mice in group 3 were intramuscularly injected with PBS and served as negative controls. The mice in Group 4 served as blank controls. One hundred μL of orbit blood were collected from each mouse. The blood samples used to prepare the sera were obtained on days 0, 7, 14, 24, and 28. Three mice from each group were sampled at random on each date. Blood were clotted and sera were prepared by centrifugation and were stored at −20 °C for later analysis. To measure serum IgG antibody, all titers of sera were evaluated using indirect ELISA.

Two weeks after the second booster dose, the mice in groups 1–3 were inoculated intraperitoneally with 0.2 mL of bacterial suspensions (1.25 × 10^6^ cfu/mL, approximately 5 × LD_50_) obtained from an 18-h culture in THB to evaluate the protection against an *S. equi* spp. *zooepidemicus* challenge [14, 15]. Another eight mice in Group 4 were injected with 0.2 mL of sterile PBS.

### The effect of anti-rGroEL antibodies on biofilm formation by *S. equi* spp. *zooepidemicus*

The biofilm assay was performed as described by Zarankiewicz et al. [16], with modifications. A *S. equi* spp. *zooepidemicus* colony was inoculated into 5 mL of THB and was cultivated with shaking at 37 °C for 12 h (late exponential/stationary growth phase) [15]. The culture was diluted 1:100 with fresh THB broth supplemented with 1% fibrinogen and with either 1% pooled mouse anti-rGroEL serum or 1% pooled non-immunized mouse sera from day 35. After a 2 h incubation at 4 °C, 200 µL of the mixtures (10^6^ cell per well) was added to each well of a 96-well polystyrene microtiter plate. The plate was incubated at 37 °C for 24 h without shaking. A *S. equi* spp. *zooepidemicus* culture in THB medium containing 1% fibrinogen was used for the positive control. Uninoculated culture medium containing 1% fibrinogen was used for the negative control. Biofilms were quantified by crystal violet staining as described by Wang et al. [17]. The medium containing free-floating bacteria was poured off and the wells were washed three times with sterile PBS. Microtiter plate wells were then stained with 200 µL of 1% (w/v) crystal violet for 10 min and washed four times with PBS to remove unbound crystal violet dye, and then air dried for 1 h. Biofilm-adsorbed crystal violet was resolubilized by adding 200 µL 95% (v/v) ethanol of each well, and the absorbance was measured at 595 nm. All the assays were performed in triplicate.

### Statistical analysis

Statistical analyses were carried out using the GraphPad Software package. Survival data were assessed by Kaplan C Meier survival analysis and tested for significance by the log rank test. Other data were analyzed using the Student’s *t* test and the *P* values <0.05 were considered significant.

## Results

### Identification of immunoreactive proteins

**Figure 1:**
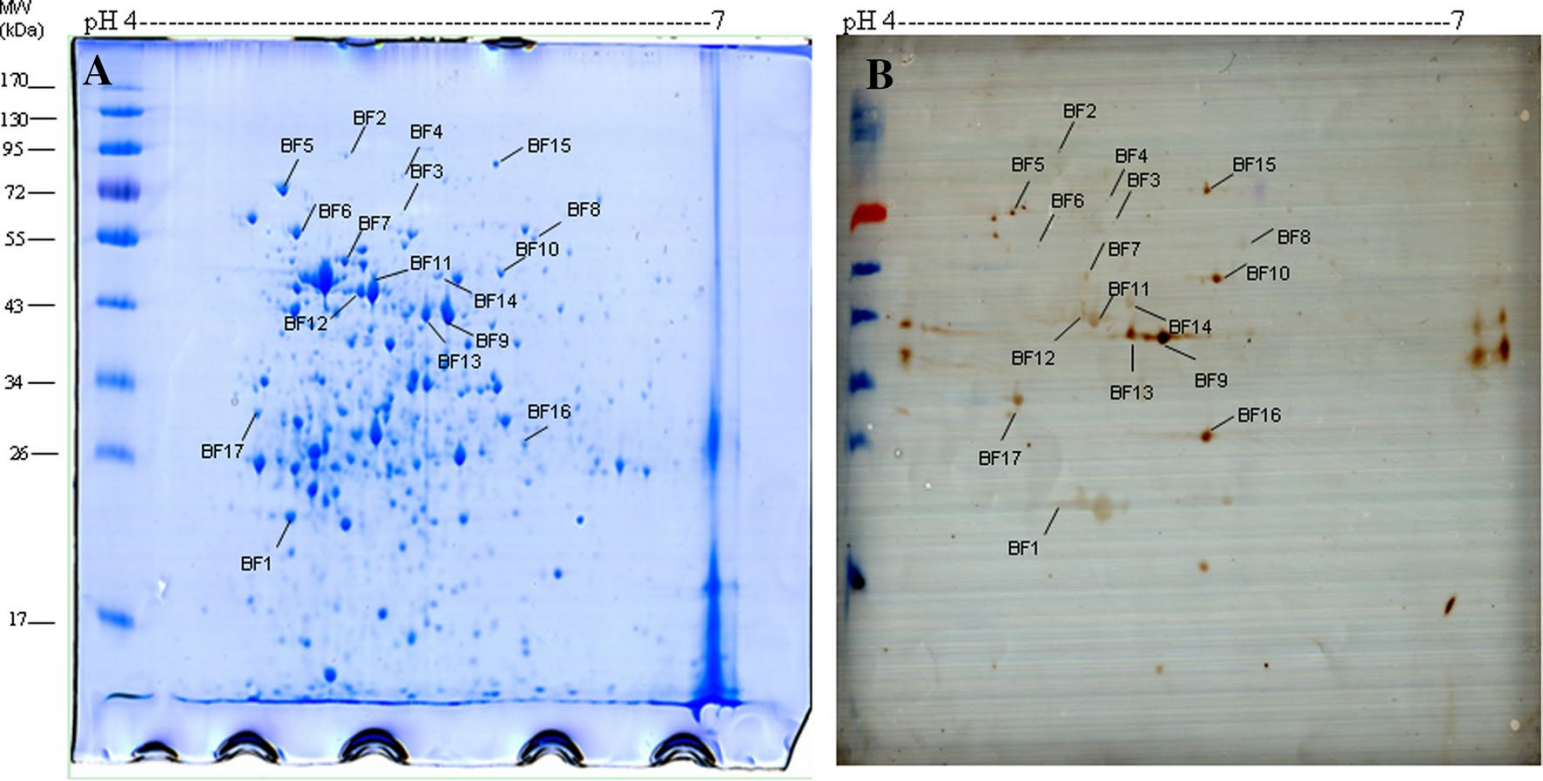
**2-D gel electrophoresis profiles of whole cell lysates of biofilm-grown** ***S. equi*** **spp.** ***zooepidemicus*** **with the immunoreactive proteins indicated.** **A** Protein staining with Coomassie B-250. **B** Western blot analysis of the immunoreactive proteins using pig convalescent serum. Immunoreactive spots are indicated by the abbreviation of biofilm (BF) followed by an arbitrary number.

A 2-DE covering a pH range of 4–7 (IPG linear gradient) was performed to separate whole cell proteins prepared from biofilm-grown *S. equi* spp. *zooepidemicus*. The spots were detected by Coomassie Brilliant Blue G-250 staining (Figure 1A). Western blotting with pig convalescent sera revealed the presence of seventeen immunoreactive proteins in the biofilm-grown bacterial samples (Figure 1B), which was consistent with our observations of the duplicated 2-D gel (Figure 1A). The seventeen spots were excised and were characterized by MALDI-TOF–MS and MALDI-TOF-TOF–MS, and the data were compared to those in the NCBI sequence database. The probability score for the match, MW, pI, and number of peptide matches were used to identify the spots. The seventeen immunoreactive spots, listed in Table 1, corresponded to seventeen different proteins. Nine of these immunoreactive proteins were also identified in planktonic-grown bacteria using the same protocol [4]: a DNA polymerase III delta subunit, elongation factor G, pyruvate kinase, transketolase, the GroEL molecular chaperone, glyceraldehyde-3-phosphate dehydrogenase, pyridine nucleotide-disulfide oxidoreductase, oligopeptide ABC transporter periplasmic oligopeptide-binding protein OppA, and elongation factor Ts. Since the GroEL molecular chaperone is essential for biofilm formation in other bacteria [18], it was selected for further analysis.

### Western blot analysis of the recombinant proteins

**Figure 2:**
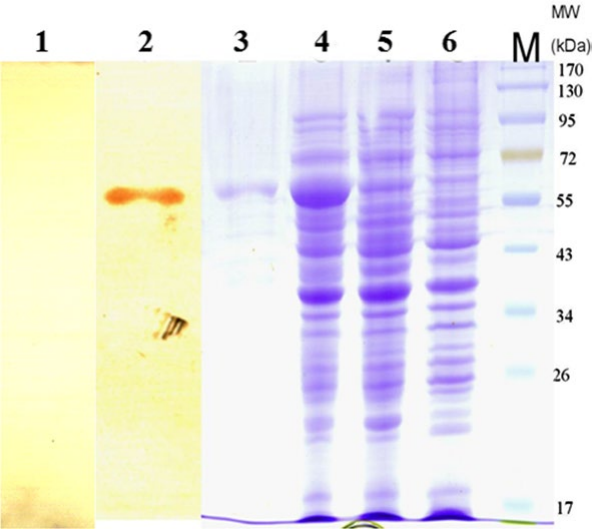
**SDS-PAGE and Western immunoblotting analysis of the rGroEL protein expressed in** ***E. coli*** **BL21.** Lane M, protein molecular mass marker; lane 1, Western blot analysis of puried rGroEL protein using pre-immune mini-pig sera as the negative control; lane 2, Western blot analysis of puried rGroEL protein using mini-pig convalescent sera; lane 3, the elution of the purified rGroEL were separated on an SDS-PAGE and stained with coomassie; lane 4, rGroEL protein in *E. coli* BL21induced with 1 mM IPTG for 4 h; lane 5, rGroEL protein without IPTG; lane 6, empty expression vector pET28a (+).

The GroEL protein of *S. equi* spp. *zooepidemicus* was cloned in *E. coli*. The immunogenicity of the rGroEL protein was confirmed by Western blotting using mini-pig convalescent sera (Figure 2).

### Antibody response to vaccination with the rGroEL protein

**Figure 3:**
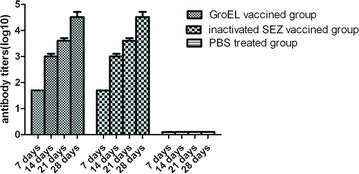
**Antibody response following vaccination.** Three mice from each group were sampled at random on each date. Data represent the mean ± standard deviation (*n* = 3 per group) of antibody titers (log 10) vs. days post-vaccination. Bars indicate standard deviations. The antibody titers of the rGroEL protein-vaccinated mice were significantly higher (*P* < 0.01) than those of the PBS-injected mice at all time points post-vaccination. No significant differences in titers were found between the rGroEL protein-vaccine and the inactivated *S. equi* spp. *zooepidemicus* vaccine (*P* > 0.05).

The GroEL-specific antibody response elicited by immunization with the rGroEL protein was monitored by determining the serum antibody titers of all the experimental mice. The GroEL protein-specific antibody titers of mice vaccinated with the recombinant protein were markedly higher than those of the PBS-injected mice at day 7 post-vaccination and continued to increase by day 28 (Figure 3). The antibody titers of the rGroEL protein-vaccinated mice were significantly higher (*P* < 0.01) than those of the PBS-injected mice at all time points post-vaccination. No significant differences in titers were found between the rGroEL protein-vaccine and the inactivated *S. equi* spp. *zooepidemicus* vaccine (*P* > 0.05).

### Immunoprotection provided by the rGroEL vaccine in mice

**Figure 4:**
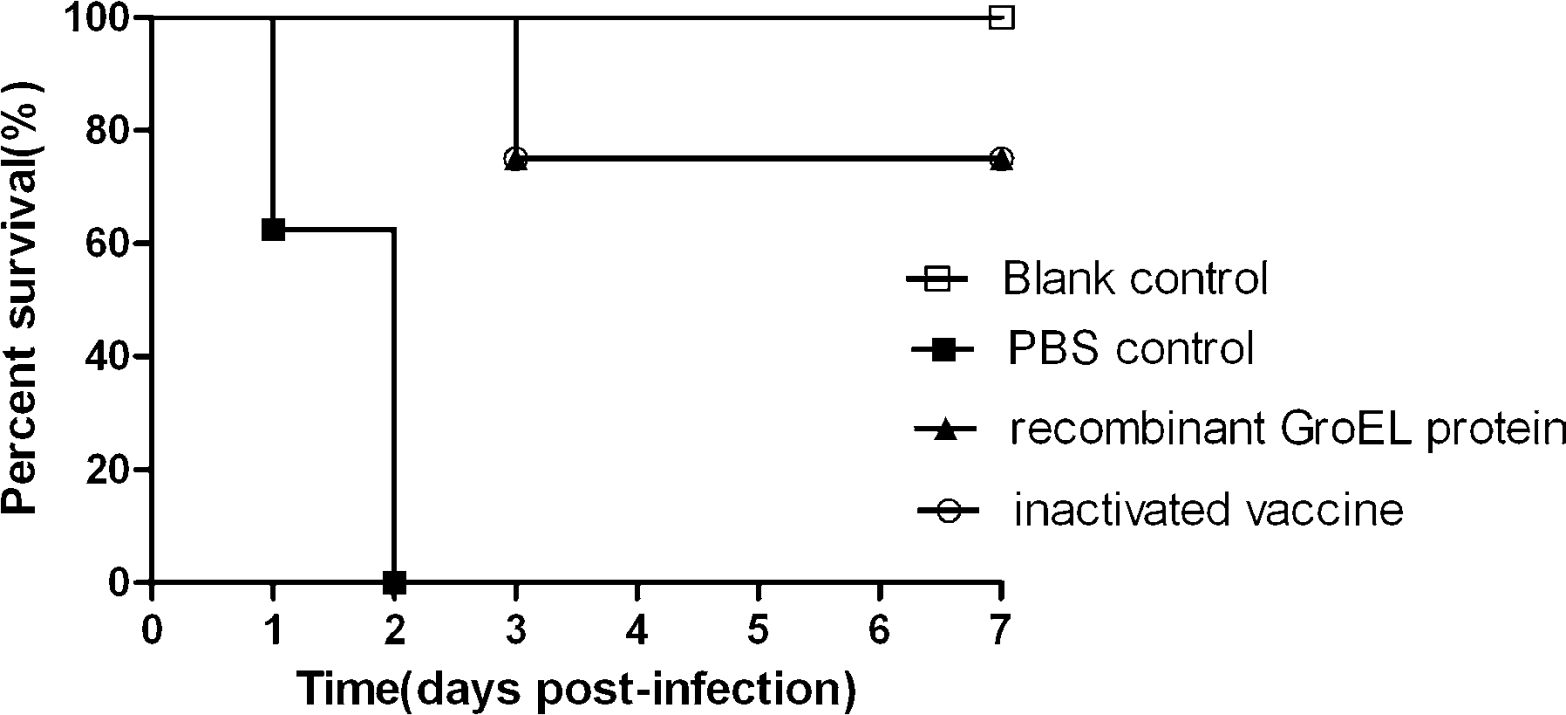
**Protective efficacy by vaccination in mice**. Mice were injected with *S. equi* *spp.* *zooepidemicus* ATCC35246 and mortality was recorded daily for 7 days. Mice in the non-immunized group died 24 to 48 h after the challenge, and the mortality rate reached 100%. Mice in the groups immunized with rGroEL or the inactivated *S. equi* spp. *zooepidemicus* vaccine died 48 to 72 h after the challenge with *S. equi* spp. *zooepidemicus* and 75% survived 7 days post-infection for these two groups. Significant differences in survival were noted, log rank test, *P* < 0.05. Compared with the non-immunized group, the mice in the groups immunized with rGroEL or the inactivated *S. equi* spp. *zooepidemicus* vaccine had higher survival rates (*P* < 0.05).

Immunized and non-immunized mice were monitored daily for 7 days following a challenge with *S. equi* spp. *zooepidemicus*. In the non-immunized group, the first death of mice occurred 24 h after the challenge, and the mortality rate reached 100% within 48 h. In the groups immunized with rGroEL or the inactivated bacterial vaccine, only 2 out of 8 mice (25%) died following the challenge with *S. equi* spp. *zooepidemicus*. More specifically, the deaths occurred at day 3 post-injection. The immunoprotection rate for these two groups was 75% in both cases (Figure 4). Compared with the non-immunized group,the mice in the groups immunized with rGroEL or the inactivated *S. equi* spp. *zooepidemicus* vaccine had higher survival rates (*P* < 0.05). No significant differences in survival rates were found between the group immunized with rGroEL and the group inactivated *S. equi* spp. *zooepidemicus* vaccine (*P* > 0.05).

### Biofilm inhibition by the anti-rGroEL antibody in vitro

**Figure 5:**
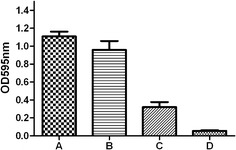
**Quantitative microtiter plate assay for biofilm production by** ***S. equi*** **spp.** ***zooepidemicus***. Biofilm formation was evaluated by monitoring the A_595 nm_ following crystal violet staining of bacterial cultures. The columns represent the means and standard deviations of three experiments. **A** Biofilm formation in THB medium (control); **B** Biofilm formation in THB medium supplemented with normal mouse serum; **C** Biofilm formation in THB medium supplemented with mouse rGroEL-antiserum; **D** THB medium. Student’s *t*-test was performed for the statistical significance analysis. Biofilm formation in THB medium supplemented with anti-rGroEL serum (**C**) was significantly lower (*P* < 0.01) than that of *S. equi* spp. *zooepidemicus* cultured in THB medium alone (**A**) or in THB medium supplemented with non-immunized pathogen-free mouse serum (**B**). No significant differences were found between *S. equi* spp. *zooepidemicus* cultured in THB medium alone and *S. equi* spp. *zooepidemicus* cultured in the THB medium supplemented with non-immunized mouse serum (*P* > 0.05).

Biofilm formation by *S. equi* spp. *zooepidemicus* was assessed in a microplate assay and crystal violet staining. Biofilm formation by *S. equi* spp. *zooepidemicus* cultured in THB medium supplemented with anti-rGroEL serum (0.32 ± 0.06) was significantly lower (*P* < 0.01) than that of *S. equi* spp. *zooepidemicus* cultured in THB medium alone (1.11 ± 0.05) or in THB medium supplemented with non-immunized pathogen-free mouse serum (0.96 ± 0.10). No significant differences in A_595 nm_ were found between *S. equi* spp. *zooepidemicus* cultured in THB medium alone and *S. equi* spp. *zooepidemicus* cultured in the THB medium supplemented with non-immunized mouse serum (*P* > 0.05). These results suggest that other components found in blood serum had no inhibitory effect on *S. equi* spp. *zooepidemicus* growth or biofilm production and that the anti-rGroEL antibody had an inhibitory effect on biofilm production (Figure 5).

## Discussion

*S. equi* spp. *zooepidemicus* is associated with a wide variety of infections in many animal species, including horses, cows, swine, sheep, and dogs [19]. More specifically, *S. equi* spp. *zooepidemicus* is the main bacterial swine pathogen in China [1]. It could also be detected in pigs without apparent clinical signs or symptoms of infection. It has also been reported to cause zoonotic infections in humans [20]. The disease is generally sporadic and outbreaks are usually of short duration; however in large herds the bacteria may be present for longer periods [21–24]. Previous studies have shown that *S. equi* spp. *zooepidemicus* can be isolated from previously described outbreaks in pig herds, thus suggesting that some *S. equi* spp. *zooepidemicus* strains can form dormant or persister cells that can be reactivated and cause infections [23, 25].

In other bacterial species, biofilms have been shown to play a key role in causing chronic infections [26, 27]. Grenier et al. [28] reported that a *Streptococcus suis* serotype 2 strain isolated from a case of meningitis in pigs could form a dense biofilm and suggested a correlation between biofilm formation and the establishment of infection. Bacteria with a capacity to colonize the host by forming biofilms have significant advantages in establishing persistent infections [29]. *S. equi* spp. *zooepidemicus* has been previously reported to form biofilms; differences in gene expression and protein profiles for planktonic- and biofilm-grown bacteria were demonstrated [5]. Attempts to efficiently control *S. equi* spp. *zooepidemicus* infections are complicated due to a lack of thorough knowledge on protective bacterial antigens. Therefore, it is important to identify antigenic components of interest for the development of a potential vaccine candidate against *S. equi* spp. *zooepidemicus* infections.

In this study, we used an immunoproteomic approach to search for immunoreactive *S. equi* spp. *zooepidemicus* proteins in biofilms. While previous studies have assessed the immunogenicity of *S. equi* spp. *zooepidemicus* using planktonic cells [3, 4], our report is the first to describe biofilm-specific proteins recognized by host antibodies. We identified seventeen immunoreactive proteins, of which nine were present in both planktonic and biofilm-grown bacteria. To our knowledge, this is the first time that the other eight immunoreactive proteins have been described in *S. equi* spp. *zooepidemicus*. The common immunoreactive proteins may be promising candidates for the development of a vaccine aimed at preventing both biofilm formation and acute *S. equi* spp. *zooepidemicus* infections. In this regard, the GroEL protein may be of high interest.

GroEL belongs to the chaperonin family of molecular chaperones. It is essential for biofilm formation in the Gram negative actinobacterium *Haemophilus influenzae* [18]. GroEL in *Streptococcus pneumoniae* can induce high antibody titers, promote lymphocyte proliferation, and induce both humoral and cell-mediated immune responses, suggesting that it may represent a protein of therapeutic interest [30]. A number of immunoproteomic studies have shown that GroEL is an immunogenic protein in a wide variety of bacteria, including *Helicobacter pylori*, *Riemerella anatipestifer*, *Brucella* spp., and *E. coli* [31–36]. Immunization with GroEL has also been shown to induce protection in a number of infection models. For instance, a significantly higher antibody titer was produced when mice were immunized with GroEL from *Streptococcus pneumoniae*, providing 50% protection rate against lethal infections [30]. In BALB/c mice, passive immunization with GroEL from *Bacillus anthracis* conferred 100% protection against *Bacillus anthracis* infections [37].

A mouse model was used to investigate the immunogenicity and protective immune response of the recombinant *S. equi* spp. *zooepidemicus* GroEL protein. Based on the ELISA results, the protein elicited high antibody titers. Animal challenge experiments with the *S. equi* spp. *zooepidemicus* ATCC35246 strain showed that 75% of the immunized mice were protected, a protection rate comparable to that obtained with inactivated bacterins.

GroEL is present in several bacterial fractions, including the cytosol, cell membrane, and extracellular material [38]. It is also the compound in the biofilm supernatant that is responsible for the anti-inflammatory effect of suppressing TNF-α production in *Lactobacilli* [39]. The GroEL of *S. equi* spp. *zooepidemicus* appeared to be involved in the production of biofilm since the presence of anti-rGroEL antibodies in the culture medium inhibited biofilm formation. The GroEL may thus be a promising target for the prevention/treatment of *S. equi* spp. *zooepidemicus* biofilm-related infections. Similar results with antibody-mediated strategies to prevent biofilm formation have been published previously. Zarankiewicz et al. [16] reported that the presence of anti-rHsp60 (GroEL) antibodies in BHI broth medium inhibited biofilm production by *Histophilus somni* in vitro. They observed small biofilm particles in the presence of the anti-rHsp60 antibody, whereas large biofilm complexes were produced in the control cultures.

In conclusion, the present study identified immunoreactive proteins in *S. equi* spp. *zooepidemicus* grown in biofilms for the first time. It also brought strong evidence that the rGroEL protein is a promising candidate for the development of vaccines against *S. equi* spp. *zooepidemicus* infections.
